# Application of Wearables to Facilitate Virtually Supervised Intradialytic Exercise for Reducing Depression Symptoms

**DOI:** 10.3390/s20061571

**Published:** 2020-03-12

**Authors:** He Zhou, Fadwa Al-Ali, Gu Eon Kang, Abdullah I. Hamad, Rania A. Ibrahim, Talal K. Talal, Bijan Najafi

**Affiliations:** 1 Interdisciplinary Consortium on Advanced Motion Performance (iCAMP), Michael E. DeBakey Department of Surgery, Baylor College of Medicine, Houston, TX 77030, USA; he.zhou2@bcm.edu (H.Z.); gueon.kang@bcm.edu (G.E.K.); 2 Fahad Bin Jassim Kidney Center, Department of Nephrology, Hamad General Hospital, Hamad Medical Corporation, PO Box 3050 Doha, Qatar; falali1@hamad.qa (F.A.-A.);; 3 Diabetic Foot and Wound Clinic, Hamad Medical Corporation, PO Box 3050, Doha, Qatar; ttalal@hamad.qa

**Keywords:** exergame, depression, hemodialysis, intradialytic exercise, end-stage renal disease, wearable, digital health, virtual reality, sensor

## Abstract

Regular exercise can reduce depression. However, the uptake of exercise is limited in patients with end-stage renal disease undergoing hemodialysis. To address the gap, we designed a gamified non-weight-bearing intradialytic exercise program (exergame). The intradialytic exergame is virtually supervised based on its interactive feedback via wearable sensors attached on lower extremities. We examined the effectiveness of this program to reduce depression symptoms compared to nurse-supervised intradialytic exercise in 73 hemodialysis patients (age = 64.5 ± 8.7years, BMI = 31.6 ± 7.6kg/m^2^). Participants were randomized into an exergame group (EG) or a supervised exercise group (SG). Both groups received similar exercise tasks for 4 weeks, with three 30 min sessions per week, during hemodialysis treatment. Depression symptoms were assessed at baseline and the fourth week using the Center for Epidemiologic Studies Depression Scale. Both groups showed a significant reduction in depression score (37%, *p* < 0.001, Cohen’s effect size *d* = 0.69 in EG vs. 41%, *p* < 0.001, *d* = 0.65 in SG) with no between-group difference for the observed effect (*p* > 0.050). The EG expressed a positive intradialytic exercise experience including fun, safety, and helpfulness of sensor feedback. Together, results suggested that the virtually supervised low-intensity intradialytic exergame is feasible during routine hemodialysis treatment. It also appears to be as effective as nurse-supervised intradialytic exercise to reduce depression symptoms, while reducing the burden of administrating exercise on dialysis clinics.

## 1. Introduction

An estimated 2.62 million people worldwide rely on hemodialysis treatment to sustain their lives [1]. Depression, likely due to the stress, exhaustion and hopelessness of hemodialysis [2,3], is the most common mental health problem among this patient population [4,5]. Depression in hemodialysis patients is an independent risk factor of cognitive–motor impairment, hospitalization, health care cost, low quality of life, and mortality [6,7,8,9,10]. Thus, there has been a growing clinical interest in treating depression in hemodialysis patients [11].

Although pharmacological treatments, such as antidepressants, are considered effective in treating depression in the general population [12], its therapeutic efficacy in hemodialysis patients is not well established, primarily due to patient safety reasons [13,14]. Instead, exercise therapy has been considered as an appropriate option to reduce depression in hemodialysis patients [15,16,17,18,19]. Scientists have demonstrated that the release of beta-endorphins during exercise plays an important role in the construction of hippocampal neurons and reduce depression [20]. In addition, exercise also reduces depression by changing the growth hormones and cortisol hormones [20]. 

However, due to the poor physical condition and medical complexity of the hemodialysis population, the approach and intensity of exercise should be carefully designed [21]. Conventional exercise strategies, including physiotherapy, aerobic exercise, and strength exercise, are not well suited for hemodialysis patients because even moderate exercise intensity can easily overtax them [21,22]. In addition, there are three main factors limiting the adherence of conventional exercise among hemodialysis patients: lack of time availability, post-dialysis fatigue or nausea, and limitation of transportation to exercise programs, which are usually offered in rehabilitation departments or cardiovascular centers but not in nephrology departments or regular dialysis clinics [23]. Because hemodialysis treatment often leaves patients feeling fatigued and, thus, reluctant to engage in physical activity, any exercise outside of the dialysis clinic may not be practical. Therefore, although previous studies have demonstrated the effectiveness of using conventional exercise strategies to reduce depression symptoms in hemodialysis patients, these studies suffer from small sample sizes (less than 15 participants) [15,16,18], low adherence rates (dropout rate > 30%) [15,16], and adverse events during exercise [15].

Hemodialysis patients are required to visit dialysis clinics three times per week and spend 4 h each time sitting or lying on a bed to receive the hemodialysis treatment. This facilitates a perfect opportunity to provide low-intensity non-weight-bearing intradialytic exercise for this patient population. Several studies have demonstrated the feasibility and effectiveness of non-weight-bearing intradialytic exercise programs to reduce depression symptoms [17,19,20]. In these studies, exercise trainers or nursing staff were required to be present during the whole exercise process to give instructions and guide the patient. However, in a real-world non-research situation, it is impractical to have an exercise trainer present in the dialysis clinic to guide each patient through the exercise. Having nursing staff administrate each exercise session for each hemodialysis patient is also impractical, which could easily overload already overburdened nursing staffs in dialysis clinics.

Recent advances in wearable technology have opened new opportunities to design virtually supervised exercise which can be applied anytime and anywhere [24,25,26]. To address the gaps described above, we have developed a low-intensity game-based non-weight-bearing lower extremities exercise program we called exergame, which can be performed during the hemodialysis treatment inside a regular dialysis clinic. The intradialytic exergame program can automatically guide the patient through the exercise process, which does not require the continuous attention of a nursing staff. In addition, by using wearable sensors and an interactive interface, the intradialytic exergame can provide gamification and visual-audio feedbacks during the exercise. This may add entertainment features into the exercise and potentially increase patient’s motivation and adherence to exercise tasks. The primary aim of this study was to evaluate the feasibility, acceptability and effectiveness of this novel intradialytic exergame program in reducing depression symptoms among patients undergoing hemodialysis and compare it to a nurse-supervised intradialytic exercise program with similar exercise tasks. We hypothesized that (1) the non-weight-bearing intradialytic exergame would be feasible and practical among hemodialysis patients; (2) both the wearable sensor-based intradialytic exergame and nurse-supervised intradialytic exercise program would be efficient to reduce depression symptoms in hemodialysis patients.

## 2. Materials and Methods

### 2.1. Study Population

This study is a secondary analysis of a clinical trial focused on examining the benefit of intradialytic exercise in adult hemodialysis patients (ClinicalTrials.gov Identifier: NCT03076528). The clinical trial was offered to all eligible hemodialysis patients who visited the Fahad Bin Jassim Kidney Center (Hamad Medical Corporation, Doha, Qatar) for hemodialysis treatment. To be eligible, the participant should be 50 years or older, be diagnosed with diabetes and end-stage renal disease that requires hemodialysis and have the capacity to consent. Participants were excluded if they had severe uncorrected visual impairment (based on the judgement of the investigator) or severe cognitive impairment (a mini-mental state examination score less than 16), which could limit the ability to interact with the intradialytic exergame interface; had major amputation; were non-ambulatory or had severe gait or balance problems (e.g., unable to walk a distance of 15 m independently with or without assistive devices or unable to stand still without moving their feet); had active foot ulcers or active infection; had a major foot deformity (e.g., Charcot neuroarthropathy); had changes in psychotropic or sleep medications in the past 6 weeks; had taken any antidepressant agents in the past 6 weeks; were in any active intervention (e.g., exercise intervention); had any clinically significant medical or psychiatric conditions; or were unwilling to participate. All participants signed a written consent form approved by the Institutional Review Board at the Hamad Medical Corporation in Doha, Qatar. All participants were randomly assigned into either an exergame group (EG) or a supervised exercise Group (SG). The randomization was done using a computer-generated list (Matlab code) with the ratio of EG vs. SG being 1:1. For the final data analysis, we only included those who had both valid baseline and fourth week depression data. 

### 2.2. Demographics and Clinical Data

Participants’ demographics and health information including age, sex, height, weight, body-mass-index (BMI), duration of hemodialysis, fall history, and number of prescription medicines were collected. All participants underwent clinical assessments, including a mini-mental state examination (MMSE) [27], short falls efficacy scale—International (FES-I) [28], vibration perception threshold test (VPT) [29], ankle brachial index test (ABI) [30] and glycated hemoglobin test (HbA1c) [31]. A cutoff FES-I score of 11 or greater was used to identify participants with high concerns about falling [28]. Plantar numbness was evaluated by VPT, measured on three plantar regions of interest in each foot: great toe, fifth metatarsal and heel. A participant was designated with diabetic peripheral neuropathy (DPN) if the maximum VPT value reached 25 volts or greater [29]. The ABI was calculated as the ratio of the systolic blood pressure measured at the ankle to the systolic blood pressure measured at the upper arm. A participant was designated with peripheral artery disease (PAD) if the measured ABI value was either greater than 1.2 or less than 0.8 [30].

### 2.3. Assessment of Depression Symptom

Depression symptoms were assessed using the Center for Epidemiologic Studies Depression (CES-D) Scale [32]. As suggested by the previous literature, a cutoff score of 16 or greater in CES-D was used to identify participants at risk for clinical depression [32].

### 2.4. Intervention: Virtually Supervised Exercise using Intradialytic Exergame

**Figure 1:**
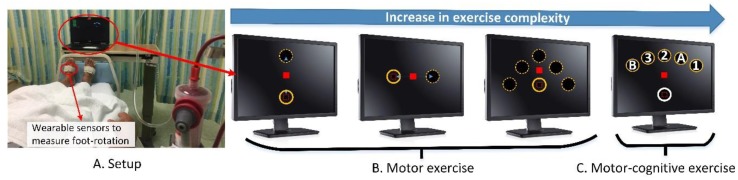
(**A**) A patient performing the intradialytic exergame during hemodialysis treatment in a regular dialysis clinic. Sensors attached on feet measure three-dimensional foot rotation. The measured foot rotation is transformed into movement of a cursor displayed on a laptop placing in front of the participant; (**B**) An illustration of the point-to-point foot rotation exercise. The participant is asked to rotate his/her foot to bring the laptop cursor (red square) into target circles as fast as possible. Exercise tasks require up to 30 degrees dorsiflexion (to move the cursor upward), plantarflexion (to move cursor downward), and medial/lateral rotation (to move the cursor to right/left). The exercise starts with simple complexity (up and down). Then the exercise complexity is gradually increased. (**C**) An illustration of the foot rotation and working memory exercise. For this exercise, in addition to the motor exercise (foot rotation), participants need to hit target circles with sequential orders (1->A->2->B->3). This exercise is considered as a dual-task exercise (motor–cognitive exercise).

The EG performed the proposed four-week intradialytic exergame program under non-weight-bearing conditions during their routine hemodialysis treatment, for three sessions per week (for total of 12 exercise sessions), which were 30 min long, including breaks. Validated inertial sensors (LEGSys^TM^, BioSensics, MA, USA) were used to estimate three-dimensional joint angles needed for providing real-time feedback during the intradialytic exercise. Sensors were attached on the top of each foot of the participants in the EG using elastic straps (Figure 1A). Each LEGSys module consists of a triaxial accelerometer (±2 g) and a triaxial gyroscope (±2000 deg/s). It can estimate real-time three-dimensional foot rotation, including inversion/eversion and dorsiflexion/plantarflexion, using the Kalman filter and quaternion approach described in detail in our previous studies [33,34,35]. Sensor data were acquired at the sampling rate of 100Hz and transmitted to an interactive interface we designed and installed on a standard laptop. The measured foot rotations from the sensor were mapped to the displacement of a laptop cursor. By rotating their foot, the participant could navigate the laptop cursor to execute a simple reaching task game (Figure 1A). Participants were instructed to rotate their feet to navigate the cursor on the laptop screen to target circles that appeared on the same screen. Exercise tasks started with simple point-to-point foot rotation in dorsiflexion and plantarflexion (navigating the cursor up and down). Later, more complex exercise tasks were provided, which required complex movement of the foot, including medial/lateral rotation with different angles as well as motor–cognitively challenged exercise such as the foot rotation and working memory exercise described in detail elsewhere [36,37,38]. Two short videos of a patient performing the intradialytic exergame can be found in Supplementary Materials.

The point-to-point foot rotation exercise was a motor exercise that required accurate mapping of foot rotation to the displacement of the laptop cursor. For the point-to-point foot rotation exercise, only the cursor (red square) and one target (solid circle) presented on the screen each time (Figure 1B). After a visual start signal, the participant was instructed to navigate the red cursor into the target circle by rotating his/her foot. If the participant successfully completed a task (i.e., moved and stopped the cursor within the center of the target circle), the target circle disappeared, followed by the appearance of a new target circle at another location (the dash circle in Figure 1B). Then, the participant repeated the task. For each task, the participant was required to rotate his/her foot up to 30 degrees (equivalent to the distance between target circles) as fast as possible. If this amount of foot rotation (30 degrees) was too difficult for the participant (e.g., the participant’s foot was too rigid), the intradialytic exergame program provided adjustable difficulty levels by increasing the mapping scale. With the largest mapping scale, the participant only needed to rotate his/her foot up to 10 degrees to navigate the cursor into target circles. Similarly, if the exercise was too simple for the participant, the mapping scale could be reduced to force the participant rotating his/her foot up to 40 degrees to complete the task. The participant was expected to compete a task rapidly (<2-s). Upon completing a task in less than 2-s, the participant was rewarded with visual (the circle exploded) and audio (positive sound) feedbacks. If the participant moved too slowly (>2-s), the participant would receive a visual feedback informing the slow execution of the task (i.e., the color of the target circle changed from yellow to green). 

The foot rotation and working memory exercise (dual-task exercise) was a motor–cognitive exercise that requires accurate coordination of foot motion while performing a cognitive task. For this exercise, a total of six circles appeared on the screen: one home circle in white and five target circles in yellow (Figure 1C). The target circles were located in a fanwise position in front of the home circle. Each target circle was marked with a number or letter (“1”, “2”, “3”, “A”, or “B”) in a randomized order. The exercise began with the cursor (red square) positioning in the home circle. The participant was instructed to navigate the cursor from the home circle to the center of a target circle in the alternative order of the numbers and letters. Specifically, the participant navigated the cursor from the home circle to the center of the first target circle that had number “1” inside. Next, the participant navigated the cursor back to the home circle, and then to the second target circle that had the letter “A” inside. The participant repeated these tasks in the order of “home”, “1”, “home”, “A”, “home”, “2”, “home”, “B”, “home”, “3”. If the participant navigated the cursor to a correct target circle, the target circle would turn red and explode with a positive rewarding sound. If the participant navigated the cursor to a wrong target circle, visual and audio feedback would be provided indicating the mistake. If the participant made a mistake, the participant would be instructed to go back to the home circle and re-do the task until successful completion. If the participant made three consecutive mistakes, a visual cue (a flashing target circle) would appear to inform the correct order.

### 2.5. Comparative Intervention: Nurse-Supervised Intradialytic Exercise

Participants randomized into the SG were also receiving hemodialysis for three sessions per week. During each hemodialysis session, a nursing staff instructed the participant in the SG to participate in a 30 min non-weight-bearing foot rotation intradialytic exercise program (including breaks) without any technology. The nurse-supervised intradialytic exercise program contained exercise tasks and intensity similar as the intradialytic exergame. However, it did not provide any game-feature or visual-audio feedback. Instead of the interactive interface, the nursing staff guided the participant throughout the intradialytic exercise program. The nurse-supervised intradialytic exercise program also lasted 4 weeks (for total of 12 exercise sessions).

### 2.6. User Experience

We evaluated the user experience of the intradialytic exergame program using a revised technology acceptance model (TAM) suggested by Schwenk et al. [39] with some modifications relevant to the scope of this study. The revised TAM questionnaire consisted of eight items, in order to evaluate perceived ease of use, perceived safety, perceived benefit, and attitude toward the use of the program. Each item was rated using a five-level Likert scale (0 = completely disagree; 1 = disagree moderately; 2 = neutral; 3 = agree moderately; 4 = absolutely agree). 

### 2.7. Statistical Analysis

All continuous data were presented as mean ± standard deviation. All categorical data were expressed as counts (percentages). A Shapiro–Wilk test was applied for testing the normality of the data. Between-group differences for continuous demographics and clinical data were compared using an independent t test. Between-group differences for categorical demographics and clinical data were compared using a Chi-square test. The effect of intradialytic exercise on depression symptoms was evaluated using repeated measures comparing the groups’ CES-D scores between pre- (i.e., baseline) and post-exercise (i.e., 4 weeks). The effect size to discriminate between pre- and post-exercise was estimated for each group using Cohen’s *d* effect size [40]. The correlation between baseline CES-D score and reduction in CES-D score from pre- to post-exercise was evaluated using Pearson’s correlation coefficient. The correlation coefficient was also interpreted as the effect size [40,41]. A two-sided *p* < 0.050 was considered to be statistically significant. All statistical analyses were performed using IBM SPSS Statistics 25 (IBM, Chicago, IL, USA).

## 3. Results

Eighty-one participants satisfied the inclusion and exclusion criteria of this study. However, the CES-D data was available and valid for only 73 participants at both baseline and 4 weeks. The analysis of demographics and clinical data for the remaining participants was summarized in Table 1. After the randomization, 37 participants were assigned into the EG, and 36 were assigned into the SG. Between the EG and SG, no difference was observed for age, sex, height, weight, BMI, duration of hemodialysis, MMSE score, FES-I score, prevalence of high concern about falling, fall history, number of prescription medications, plantar sensation, prevalence of DPN, prevalence of PAD, or HbA1c value (all *p* > 0.050).

All participants in the EG and SG completed all exercise sessions of the four-week program. No adverse events related to the intradialytic exergame or foot rotation exercise were reported. Table 2 showed the descriptive results of the user experience TAM questionnaire in mean, standard deviation, median, and range. The majority of the EG participants “absolutely agreed” (i.e., rated 4 out of 4 in the Likert-scale) that they had fun while exercising (mean score = 3.22) and experienced no problems or safety concerns (mean score = 3.56). The sensor feedback helped the majority of the EG participants learn the exercises quickly (mean score = 3.67). The majority of the EG participants agreed that the form and design of the technology was optimal (mean score = 3.50). Most participants disagreed that exercises were too fast or exhausting (mean score = 0.25). Participants disagreed moderately that movements were difficult to perform (mean score = 0.86).

**Figure 2:**
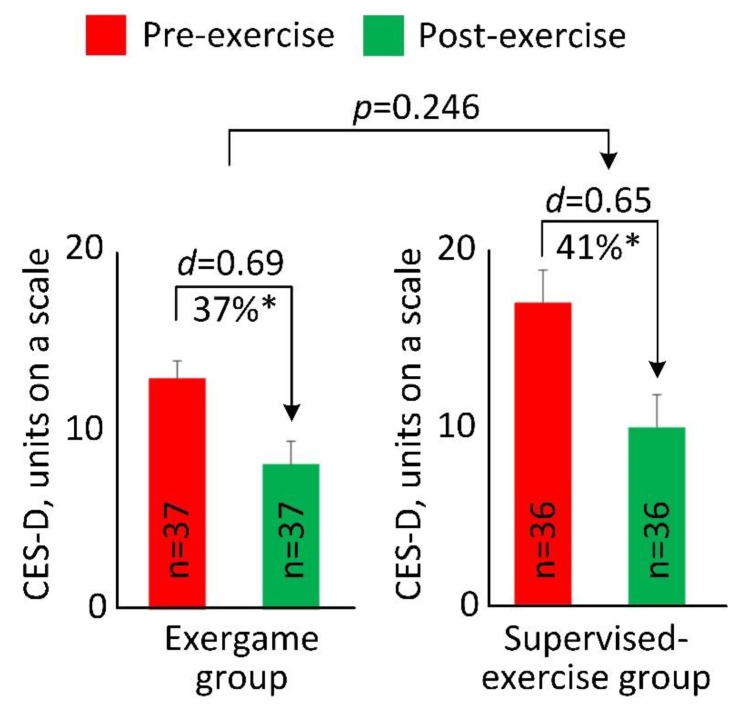
Pre- and post-exercise Center for Epidemiologic Studies Depression (CES-D) scale score for the exergame group and the supervised exercise group. The error bar represents the standard error. “*n*” denotes number of participants per group. “*d*” denotes Cohen’s *d* effect size calculated between pre- and post-exercise for each group. “*” denotes when the pre- and post-exercise comparison achieved a statistically significant level (*p* < 0.050).

At the baseline of the intradialytic exercise program, 27 participants (37%) were identified at risk for clinical depression (CES-D≥16) in the two groups. At the end of the intradialytic exercise program, the number of participants at risk for clinical depression in the EG reduced by approximately 36% (*n* = 11 for pre-exercise; *n* = 7 for post-exercise), and the number of participants at risk for clinical depression in the SG reduced by approximately 43% (*n* = 16 for pre-exercise; *n* = 9 for post-exercise). Figure 2 illustrated changes in the CES-D score between pre- and post-exercise for both groups. After 4 weeks of intradialytic exercise, compared to baseline, the CES-D score reduced from 12.9 ± 6.0 to 8.1 ± 7.8 in the EG (37% reduction, *p* < 0.001, *d* = 0.69), and from 16.8 ± 11.0 to 9.9 ± 10.5 in the SG (41% reduction, *p* < 0.001, *d* = 0.65). There was no significant difference in reduction of CES-D score between the EG and SG (*p* = 0.246).

**Figure 3:**
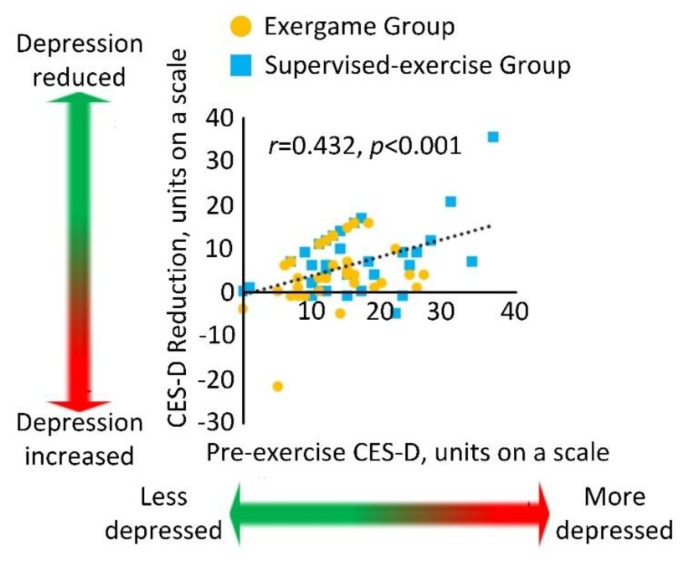
Correlation between the baseline CES-D score and the score reduction for the exergame and supervised exercise groups.

Figure 3 demonstrated the correlation between the baseline CES-D score and the score reduction for both the EG and the SG. A significant correlation with medium effect size was observed, indicating that participants with higher CES-D score at baseline had more of a reduction in CES-D score at 4 weeks (*r* = 0.432, *p* < 0.001).

## 4. Discussion

To our knowledge, this is the first study that used wearable technology to provide a game-based non-weight-bearing intradialytic exercise program for hemodialysis patients. We were able to confirm our hypothesis that the proposed intradialytic exercise program is feasible and practical to be performed during routine hemodialysis treatment inside a regular dialysis clinic. It is acceptable among patients undergoing hemodialysis. In addition, this is the first study that objectively evaluated the effect of the low-intensity non-weight-bearing intradialytic exergame to reduce depression symptoms in hemodialysis patients. We have showed that virtually supervised intradialytic exercise therapy appears to be as effective as nurse-supervised intradialytic exercise therapy to reduce depression symptoms. No requirement for continuous attention of a physical therapist or a trained nursing staff for the intradialytic exergame program is however considered as an advance compared to nurse-supervised intradialytic exercise intervention. This may improve the uptake of routine intradialytic exercise intervention for hemodialysis patients, without adding significant burden to the already overburdened nursing staffs in dialysis clinics.

Comparing to the conventional exercise strategies, the advantage of the low-intensity non-weight-bearing intradialytic exercise program is that it can be performed while patients receiving routine hemodialysis treatments in regular dialysis clinics. In order to participate in the conventional physical exercise, hemodialysis patients usually need to visit out-clinic facilities on a non-dialysis day [42], which greatly limits the adherence to exercise. Moreover, for patients with severe physical and physiological issues, it is even more difficult to bring them to out-clinic facilities on a non-dialysis day for exercise [23]. In addition, due to the deconditioning in physical function [43], the conventional exercise such as cycling, walking, jogging, and resistance training may become too difficult for hemodialysis patients and easily overtax them [21,22]. On the other hand, the intradialytic exergame enables adjustable exercise tasks depending on the motor capacity and cognitive status of the patient. In this study, most participants agreed that the intradialytic exergame were not too fast nor exhausting. They did not experience any problem or safety concern. No adverse event related to the intradialytic exergame occurred. In this study, we excluded individuals with severe visual impairment or severe cognitive impairment (MMSE < 16), which could limit the ability to interact with the intradialytic exergame interface. However, we included those with mild to moderate cognitive impairment. According to our results, 23% of participants had cognitive impairment (MMSE < 24), but did not experience any problem to follow instructions and achieved to complete all exercise tasks. This demonstrated the feasibility of intradialytic exergame for those with mild to moderate cognitive impairment.

Previous studies have demonstrated effects of non-weight-bearing intradialytic exercise, such as ankle rotation, toe flexion & extension, joint warming action, stretching exercise, etc., to reduce depression symptoms. Razaei et al. provided a 35 min non-weight-bearing intradialytic exercise program three times per week for 10 weeks to 25 hemodialysis patients. At the end of the program, the depression score significantly decreased by 53% (*p* < 0.001) [19]. Santhi et al. recruited seven hemodialysis patients and provided them a 15-min non-weight-bearing intradialytic exercise program supervised by the nursing staff or the physiotherapist for 8 weeks [20]. Non-significant trends of reduction in depression, anxiety, and stress (Chi square = 0.295–1.333) were observed at the end of the exercise program [20]. In our present study, results demonstrated that our intradialytic exergame program is also efficient to reduce depression symptoms in hemodialysis patients, even with a shorter period of time (4 weeks). In addition, our results showed that hemodialysis patients with more severe depression symptoms (higher CES-D score) enjoyed more benefits from the intradialytic exergame program.

In this study, the SG also received 4 weeks (three sessions per week, 30 min per session) of non-weight-bearing foot rotation intradialytic exercise without any technology, game-feature, or visual-audio feedback, but under a supervision by nursing staffs. Comparing to the nurse-supervised intradialytic exercise, wearable and interactive interface technologies enable real-time visual and audio feedbacks in the intradialytic exergame. Hemodialysis patients can visually perceive their performances and errors during the exercise. In the intradialytic exergame, hemodialysis patients need to rotate ankle joint to certain range and achieve certain speed to complete the exercise task. This can standardize the exercise intensity and push the boundary of hemodialysis patients. The intradialytic exergame also includes game-features such as rewarding elements and animations, which could be more attractive than the non-technological foot rotation exercise. Therefore, it has the potential to increase exercise adherence and be more efficient to reduce depression symptoms [44]. Furthermore, the intradialytic exergame is virtually supervised by the computer program. It can reduce the burden of administrating exercise during hemodialysis treatment, making the exercise more feasible and practical in a regular dialysis clinic. It also has the potential to facilitate tele-rehabilitation.

A major limitation of this study is the relatively low sample size, which could be underpowered for the clinical conclusion. This study is a secondary analysis of a clinical trial focused on examining the benefit of intradialytic exergame in gait and balance among hemodialysis patients. We only performed CES-D for depression assessment in the clinical trial. The CES-D is a screening test for depression symptoms but not a sufficient tool to diagnose depression. A future study using comprehensive psychological assessments for evaluating depression is needed. Due to the same reason, we did not have a non-exercise control group in this study. However, the effectiveness of low-intensity non-weight-bearing intradialytic exercise to reduce depression symptoms has been demonstrated in previous studies [17,19,20]. In this study, we aimed to provide a sensor-based intradialytic exergame with gamifications and real-time visual-audio feedbacks, which may increase the exercise adherence and relieve the burden of administrating exercise during routine hemodialysis treatment. Higher reduction in depression symptoms was observed for those with more severe depression symptoms. Without the non-exercise control group, the “regression toward the mean” effect cannot be excluded. A future study recruiting both an intradialytic exergame intervention group and a non-exercise control group is needed. In this study, although randomization was done using a computer-generated list, the EG and SG were not perfectly randomized. The EG had lower prevalence of risk for clinical depression and lower average CES-D score than the SG at the baseline. However, the difference did not reach statistical significance, so we did not adjust the results by pre-exercise depression score. In this study, we evaluated the immediate reduction of depression score after 4 weeks of the intradialytic exergame program. However, the ideal dosing of intervention and retention of exercise benefits remain unclear. On the other hand, since hemodialysis patients have to visit the dialysis clinic three times per week until they get a kidney transplant, we envisage that the proposed intradialytic exergame could be implemented as a part of routine dialysis clinic to reduce the likelihood of depression symptoms.

## 5. Conclusions

This study demonstrated the feasibility and acceptability of the sensor-based non-weight-bearing intradialytic exergame program to be performed during routine hemodialysis treatment in a regular dialysis clinic. The sensor-based intradialytic exergame program enables delivering a personalized and virtually supervised intradialytic exercise intervention. It also appears to have similar effect as nurse-supervised intradialytic exercise therapy to reduce depression symptoms in hemodialysis patients, especially in those with severe depression symptoms. Its key advantage compared to nurse-supervised intradialytic exercise is that there is no requirement for the continuous attention of a physical therapist or trained nursing staff. It can reduce the burden of implementing exercise intervention during routine hemodialysis treatment.

## Figures and Tables

**Table 1:** Demographics and clinical data of the study population.

	Exergame Group (EG, *n* = 37)	Supervised Exercise Group (SG, *n* = 36)	*p*-Value
*Demographics*			
Age, years	62.7 ± 6.8	66.5 ± 10.0	0.060
Sex (female), *n* (%)	18 (49%)	22 (61%)	0.285
Height, m	157.1 ± 25.4	157.5 ± 10.2	0.932
Weight, kg	77.8 ± 17.2	82.4 ± 22.9	0.328
BMI, kg/m^2^	30.0 ± 6.3	33.2 ± 8.4	0.068
*Clinical data*			
At risk for clinical depression (CES-D ≥ 16), *n* (%)	11 (30%)	16 (44%)	0.193
Duration of hemodialysis, years	4.8 ± 5.0	4.0 ± 3.8	0.487
Had fall in last 12-month, *n* (%)	8 (22%)	9 (25%)	0.733
MMSE, units on a scale	27.5 ± 2.8	25.4 ± 4.9	0.052
Concern for fall (FES-I score), units on a scale	12.7 ± 5.2	14.7 ± 6.3	0.134
High concern about falling (FES-I ≥ 11), *n* (%)	19 (51%)	24 (67%)	0.184
Number of prescription medications, *n*	8 ± 3	7 ± 3	0.835
Plantar sensation (VPT), Volt	33.2 ± 17.1	33.7 ± 15.9	0.885
Diabetic peripheral neuropathy (VPT ≥ 25), *n* (%)	22 (60%)	23 (64%)	0.697
Peripheral artery disease (ABI < 0.8 or ABI > 1.2), *n* (%)	20 (54%)	25 (69%)	0.176
HbA1c, %	6.6 ± 1.6	6.9 ± 1.6	0.378

BMI: body-mass-index; CES-D: Center for Epidemiological Studies Depression; MMSE: Mini-Mental State Examination; FES-I: Short Fall Efficacy Scale-International; VPT: Vibration Perception Threshold; ABI: Ankle Brachial Index.

**Table 2:** Results of the user experience questionnaire.

Question	Mean	Standard Deviation	Median	Range
1: It was fun to use the sensor-based exercise technology	3.22	0.19	4	0–4
2: Usage of the technology was possible without problems at any time	3.56	0.13	4	1–4
3: The form and design of the technology are optimal for me	3.50	0.13	4	2–4
4: I feel more energetic at home after doing exercise	3.03	0.20	4	1–4
5: Thanks to the sensor feedback, I could quickly learn all exercises	3.67	0.10	4	2–4
6: I feel that the exercises were going too fast and exhausting me	0.25	0.12	0	0–3
7: Some of the movements were difficult to perform	0.86	0.19	0	0–3
8: I felt safe using the exercise technology	3.75	0.07	4	3–4

Answer categories: 0 = disagree completely; 1 = disagree moderately; 2 = neutral; 3 = agree moderately; 4 = agree absolutely.

## Supplementary Materials

The following are available online at https://www.mdpi.com/1424-8220/20/6/1571/s1, Two short videos of a patient performing the intradialytic exergame. https://drive.google.com/file/d/17xSRaRJidgOshXHh-_AVzeBW38svGqyy/view?usp=sharing, https://drive.google.com/file/d/1xMB63rQV_4-f5O1SP0PYXKoRZtT8t_xo/view?usp=sharing.
